# pyDockDNA: A new web server for energy-based protein-DNA docking and scoring

**DOI:** 10.3389/fmolb.2022.988996

**Published:** 2022-10-06

**Authors:** Luis Angel Rodríguez-Lumbreras, Brian Jiménez-García, Silvia Giménez-Santamarina, Juan Fernández-Recio

**Affiliations:** ^1^ Barcelona Supercomputing Center, Barcelona, Spain; ^2^ Instituto de Ciencias de la Vid y del Vino (ICVV), Logroño, Spain; ^3^ Zymvol Biomodeling SL, Barcelona, Spain; ^4^ ICMol, Universitat de València, Paterna, Spain

**Keywords:** structural modeling, *Ab initio* docking, protein-DNA interaction, scoring function, docking benchmark, nucleotide parameters

## Abstract

Proteins and nucleic acids are essential biological macromolecules for cell life. Indeed, interactions between proteins and DNA regulate many biological processes such as protein synthesis, signal transduction, DNA storage, or DNA replication and repair. Despite their importance, less than 4% of total structures deposited in the Protein Data Bank (PDB) correspond to protein-DNA complexes, and very few computational methods are available to model their structure. We present here the pyDockDNA web server, which can successfully model a protein-DNA complex with a reasonable predictive success rate (as benchmarked on a standard dataset of protein-DNA complex structures, where DNA is in B-DNA conformation). The server implements the pyDockDNA program, as a module of pyDock suite, thus including third-party programs, modules, and previously developed tools, as well as new modules and parameters to handle the DNA properly. The user is asked to enter Protein Data Bank files for protein and DNA input structures (or suitable models) and select the chains to be docked. The server calculations are mainly divided into three steps: sampling by FTDOCK, scoring with new energy-based parameters and the possibility of applying external restraints. The user can select different options for these steps. The final output screen shows a 3D representation of the top 10 models and a table sorting the model according to the scoring function selected previously. All these output files can be downloaded, including the top 100 models predicted by pyDockDNA. The server can be freely accessed for academic use (https://model3dbio.csic.es/pydockdna).

## Introduction

Proteins and nucleic acids are fundamental biological macromolecules whose functions and interactions are vital to regulating cell’s life. Their interactions regulate many biological processes such as protein synthesis, signal transduction, DNA storage, and DNA replication and repair, among others. Learning how protein and DNA interact is fundamental to fully elucidate many central biological processes and disease mechanisms, and can also support the discovery of novel therapeutic targets. Although 192,025 structures have been experimentally determined and deposited in the June 2022 release of Protein Data Bank (PDB), only 10,480 of them correspond to protein-nucleic acid complexes (this includes 6,732 protein-DNA complexes). Thus, the number of protein-DNA structures experimentally determined is clearly much smaller than the number of protein-DNA complexes that are expected to be formed in cells. This gap is partially explained by the difficulty of the experimental determination process, i.e. a very time-consuming process in the best scenarios or impossible in many cases due to limitations on the experimental techniques. For this reason, a computational approach on modelling protein-DNA interactions could be of enormous help.

Even though theoretical models of macromolecular structures are usually less accurate than direct experimental measurements, they can yield sufficient information to build a working hypothesis, complementing experimental approaches in elucidating protein-DNA interactions and guiding further experimental analyses to identify essential amino acids or nucleotide residues. From a computational point of view, there are two main approaches to model the structure of a protein-DNA complex: template-based modelling and *ab initio* docking. Template-based modelling aims to model a complex based on the structure of a homologous complex. The popularity of template-based methods has increased in the past years, especially for modelling protein-protein complexes, thanks to the development and support of many structural databases of protein interactions that can provide the required templates, such as 3D Complex (Levy et al., 2006), Dockground (Kundrotas et al., 2018), or Interactome3D (Mosca et al., 2013). However, the quality of template-based predictions clearly depends on the availability of suitable templates, not particularly high in the case of protein-DNA interactions, which makes template-based approaches of very limited applicability for protein-DNA modeling. On the other hand, *ab initio* docking methods aim to predict the three-dimensional structures of macromolecular complexes, starting from the atomic coordinates of their components. *Ab initio* docking methods do not depend on available structural data for homologous complexes, which makes them more useful in the actual protein-DNA context.

The methodology for the prediction and modelling of protein-protein complexes is very well established despite there are still many challenges to be addressed. Numerous protein-protein docking methods have been developed and assessed as shown in the Critical Assessment of PRediction of Interactions (CAPRI) community-wide experiment. During the past editions of the CAPRI experiment (Janin et al., 2003), targets other than protein-protein complexes were proposed: protein-RNA complex (Lensink and Wodak, 2010) (T33, T34), protein-peptide (T60-64) or protein-heparin (T57) among others. However, protein-DNA docking received limited attention from the CAPRI community and developers of computational methods. Macromolecular docking protocols that accept protein and DNA coordinates as input include FTDock (Gabb et al., 1997), GRAMM-X (Tovchigrechko and Vakser, 2006), HEX (Macindoe et al., 2010), PatchDock (Schneidman-Duhovny et al., 2005; Macindoe et al., 2010) and NPDock (Tuszynska et al., 2015), as well as HDock (Yan et al., 2017), ClusPro (Comeau et al., 2004) and HADDOCK (Van Zundert et al., 2016) servers. From this list of tools, only NPDock and HDock were originally developed for protein-nucleic acid docking; the rest were developed as protein-protein docking tools that can also accept nucleic acids coordinates, but they lack an intrinsic scoring function dedicated to assessing protein-DNA interactions. These protocols usually report high predictive rates in bound conditions, i.e. when the co-crystallized partners in a known complex structure are separated and then re-docked. However, despite bound docking is useful for testing and development purposes, it does not represent realistic conditions and thus it is of limited practical value for biology. Therefore, it is important to have available datasets to test protein-DNA docking tools in unbound conditions. Compared to protein-protein docking, where the most recent release of the Weng’s group Protein-Protein Docking Benchmark 5.5 (Vreven et al., 2015) has 257 entries, and to protein-RNA docking, where there are different reported benchmarks (Barik et al., 2012; Pérez-Cano et al., 2012; Huang and Zou, 2013; Nithin et al., 2017), for protein-DNA docking there is only one available benchmark, which contains 47 complexes (van Dijk and Bonvin, 2008). Using this benchmark, protein-DNA docking protocols report moderate success rates in unbound conditions. For instance, on a subset of 23 cases from this benchmark, HDock success rate for top 10 models (i.e. at least one near-native structure within the top 10 models) is less than 10%, while success rate for top 100 is slightly over 30% (Yan et al., 2017). NPDock reports a maximum success rate (i.e. at least one near-native conformation found in the entire prediction set) of 7/47 (15%) (Tuszynska et al., 2015). Protein-DNA docking with HADDOCK reported an excellent performance (van Dijk and Bonvin, 2010) when using restraints based on the real interface. This represents a very promising approach, but in a realistic scenario, lack of knowledge on the actual complex interface might limit its application. A more recent coarse-version of HADDOCK protein-DNA docking shows similar accuracy with ∼6-fold speed increase over atomistic calculations (Honorato et al., 2019). The need of new computational tools to address unbound protein-DNA docking is clear. We present here a new web server that implements the pyDockDNA protein-DNA docking and scoring protocol, as a new module of pyDock version 4 (upcoming publication). The original pyDock docking and scoring approach (Cheng et al., 2007), which showed excellent performance for the prediction of protein-protein docking (Lensink et al., 2019; Rosell et al., 2020), has been rewritten in *Python* 3 and extended for its application to protein-DNA docking, with new functionalities to handle the nucleic acid structures and upgraded atomic solvation parameters for a more accurate scoring of protein-DNA interactions.

## Materials and methods

### Data sets: Protein-DNA docking benchmark and external case studies

In order to test the new pyDockDNA docking protocol, we used a previously developed protein-DNA docking benchmark (version 1.2) (van Dijk and Bonvin, 2008). The benchmark contains bound and unbound x-ray crystallography and NMR structures for 47 protein-DNA complexes, in which DNA is in B-DNA conformation. These are classified as ‘easy’, ‘intermediate’ or ‘difficult’ cases, based on the interface RMSD values between the bound and unbound components of the complex.

An additional set of case studies was compiled following the criteria selection used in the above described protein-DNA docking benchmark. This test set is composed of ten protein-DNA complexes, where both bound and unbound structures are available for each reference complex, and the sequences are different from those in the first protein-DNA docking benchmark. Protein-DNA complex and unbound structures were compiled from the Protein-DNA Interface Database (PDIdb) (Norambuena and Melo, 2010) and the Protein Data Bank (PDB) (Berman et al., 2000). Only complexes that meet the following conditions were considered: 1) DNA sequence length larger than eight base pairs, and 2) proteins without mutations in the core of the complex interface. To find the protein unbound structures of the selected protein-DNA complexes, all the PDB entries containing only protein structures were retrieved, including structures solved by NMR. Crystallographic structures with a resolution worse than 3.0 Å were not considered. To avoid redundancy, entries with sequence similarity ≥90% were discarded. PDBeFOLD (Krissinel and Henrick, 2004) was used to find correspondences between bound and unbound protein structures. This tool performs structural alignments between two (pairwise alignment) or more (multi-alignment) molecules using their 3-dimensional structures. The alignment is based on the Secondary Structure Matching algorithm (Krissinel and Henrick, 2004). Alignments with a Q-score higher than 8.0, high P-score and sequence similarity around 90–100% were accepted as the corresponding unbound. Then, both bound and unbound structures for each case, were post-processed according to the protocol followed in a previously developed protein-DNA docking benchmark, for instance by checking consistency between unbound and bound coordinates in chain IDs, residue numbers and atom names (van Dijk and Bonvin, 2008). The unbound DNA models were generated by using the software 3DNA (Lu and Olson, 2003; Lu and Olson, 2008), in canonical B-DNA conformation (fiber model 4).

This additional test set (Table 1) is freely available at the “Help” section of the server (https://model3dbio.csic.es/pydockdna/info/faq_and_help#extended_benchmark).

**TABLE 1:** List of case studies.

PDB complex	Protein	PDB unbound protein	RMSD unbound-bound protein	DNA	RMSD unbound-bound DNA
5JLT	phage T4 MotA DNA-binding domain	1KAF	0.83^a^	22bp dsDNA	1.89
2X6V	TBX5	2X6V	0.55	11bp DNA	2.03
3POV	SOX	3FHD	1.46	19bp DNA	2.26
4UUV	ETV4 DNA-binding ETS domain	5ILU	1.24	10bp DNA	2.81
2NTC	sv40 large T antigen	2FUF	1.13^a^	21-nt PEN element of the SV40 DNA origin	2.96
2ITL	sv40 large T antigen	4NBP	5.37^a^	24-nt PEN element of the SV40 DNA origin	3.84
3MFK	Protein C-Ets1	1GVJ	5.61^a^	stromelysin-1 promoter DNA	4.34
2PI0	IRF-3	3QU6	0.76^a^	PRDIII-I region of human interferon-B promoter strand 1	4.46
1O3R	catabolite gene activator protein	4R8H	0.65	11bp DNA	4.77
3MLO	Ebf1	3LYR	0.71^a^	22bp DNA	5.11

a In cases with more than one protein-DNA, interface in the x-ray structure, the average value is provided.

### Sampling

In this first step, the input files with the coordinates in PDB format for the structures (or models) of a protein and a DNA molecule (which can be B-DNA or any other conformation) are checked for potential format errors. Missing side-chains in the protein are rebuilt with SCWRL 3.0 (Bower et al., 1997), and the electrostatics Amber94 force field (Cornell et al., 1995) is loaded, assigning the charges to the atoms. Then, rigid-body docking poses between the protein and the DNA, represented as 3D grids, are generated with a faster and parallelized version of the original FTDock (v2.0) software (Gabb et al., 1997) in which the number of cells in the grid is optimized for maximum computing efficiency (Jiménez-García et al., 2013). The molecule (protein or DNA) with the longest maximal distance between any pair of atoms is considered the receptor, that is, the fixed molecule, and the other one is the ligand or mobile molecule. By default, the program uses 0.7 Å grid cell size, 1.3 Å surface thickness, 12° rotation sampling, and keeps the best three poses for each rotation. For each target, a total of 10,000 docking poses are generated.

### Scoring

Finally, the protein-DNA docking poses are ranked using a scoring function composed of electrostatics, desolvation and van der Waals energy. This new pyDockDNA scoring function is adapted from the previously pyDock scoring function for protein-protein docking (Grosdidier et al., 2007; Jiménez-García et al., 2013), which now includes atom types for nucleotides from Amber94 force field (Cornell et al., 1995) in order to calculate for the modelled protein-DNA complexes. The nucleotide AMBER atom types have been mapped to the previously defined atom types in pyDock within a new parameter set (*nuc.dat*).

### Implementation of pyDockDNA web server

**FIGURE 1:**
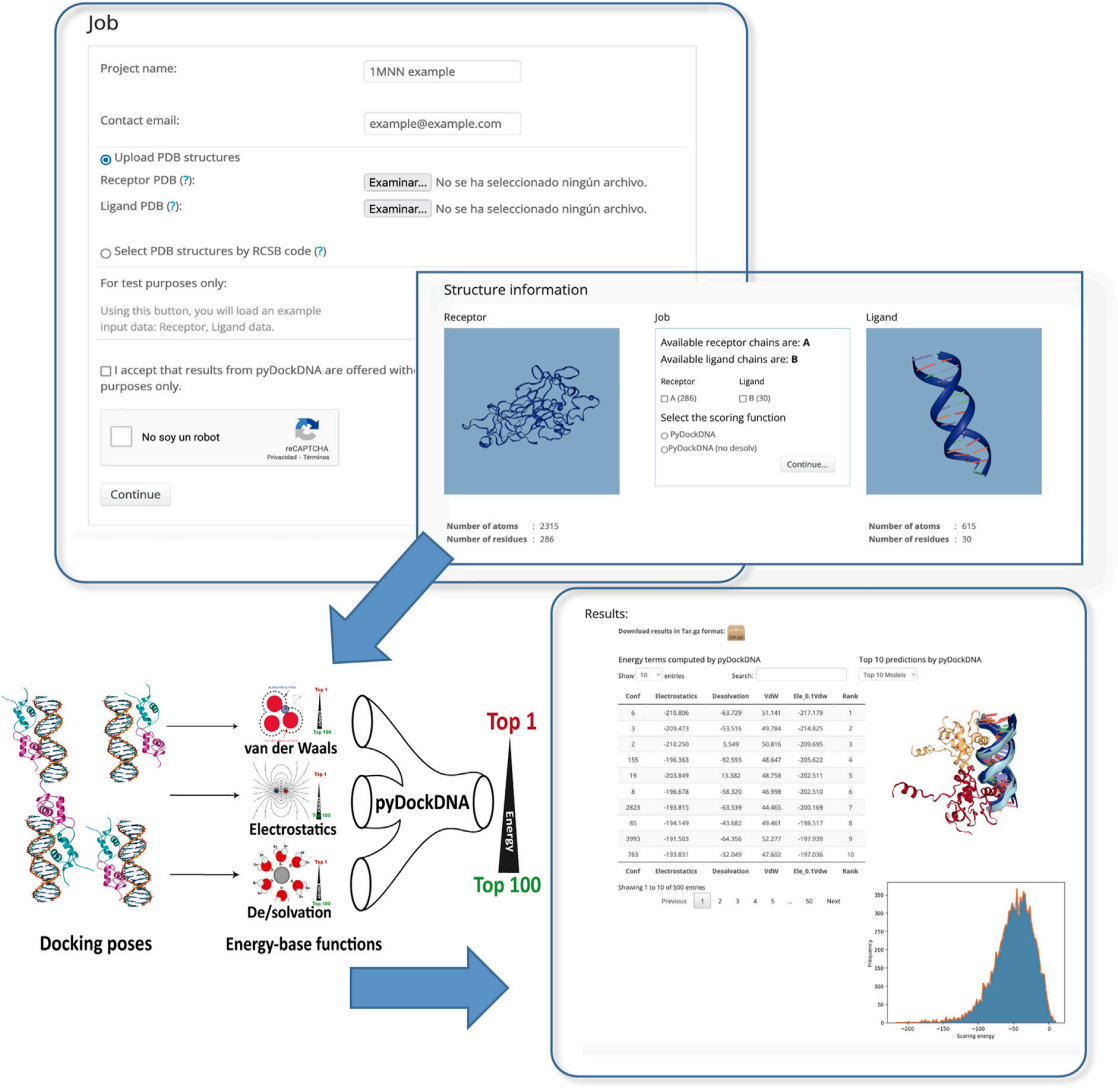
Schematic representation of the pyDockDNA web server main functionalities.

The program pyDockDNA is built as a module of the new pyDock 4.0 version (upcoming publication), thus include the same third-party programs, modules and tools from previous versions of pyDock as well as new functionalities to handle the nucleic acid structures properly. The user can select the chains to be docked, the energetic scoring function, and even include external information [from available experimental data or using predictive methods such as the DBSI server (Sukumar et al., 2016), for instance] as residue-nucleotide distance restraints to rescore docking models as previously described for pyDockRST (Chelliah et al., 2006). The output will be a set of docking models represented in different formats: 1) the 3D structure of the best-scoring 10 docking models in terms of scoring can be visualized in the output screen, 2) the PDB files for the best-scoring 100 models can be directly downloaded, and 3) the rotation/translation vectors are provided to generate up to a total of 10,000 docking poses. A summary of the docking results can be visualized as a plot with the distribution of the different energy values obtained for all docking poses (Figure 1).

### Clustering of protein-DNA docking models in benchmarking

When testing this software (see Results) we have run several docking executions in parallel, using different initial random rotations for the input structures, and the best-scoring 100 resulting models for each individual run were merged into a single pool. To avoid redundancy in the final set, all docking orientations were clustered by pyProCT analysis software (Gil and Guallar, 2014), which implements the GROMOS clustering algorithm (Daura et al., 1999). Distance matrix is built with pyRMSD with the option “QCP OMP CALCULATOR” to compute the ligand root-mean-square deviation (L-RMSD) values for all pairs of docking orientations after their receptors were superimposed (https://github.com/victor-gil-sepulveda/pyRMSD/). A cut-off value of 4.0 Å was used for L-RMSD to define the clusters. For each defined cluster of models, the orientation with the lowest docking score is selected as the cluster representative.

### Docking performance

We have evaluated the predicted performance of pyDockDNA in different conditions based on the success rates for the obtained top *N* docking models, which is the % of benchmark cases in which a near-native (acceptable) solution is found within the top *N* docking models. A near-native solution is defined as a docking orientation model with L-RMSD ≤ 10 Å with respect to the reference structure.

## Results and Discussion

### Performance of pyDockDNA evaluated on the protein-DNA docking benchmark

**FIGURE 2:**
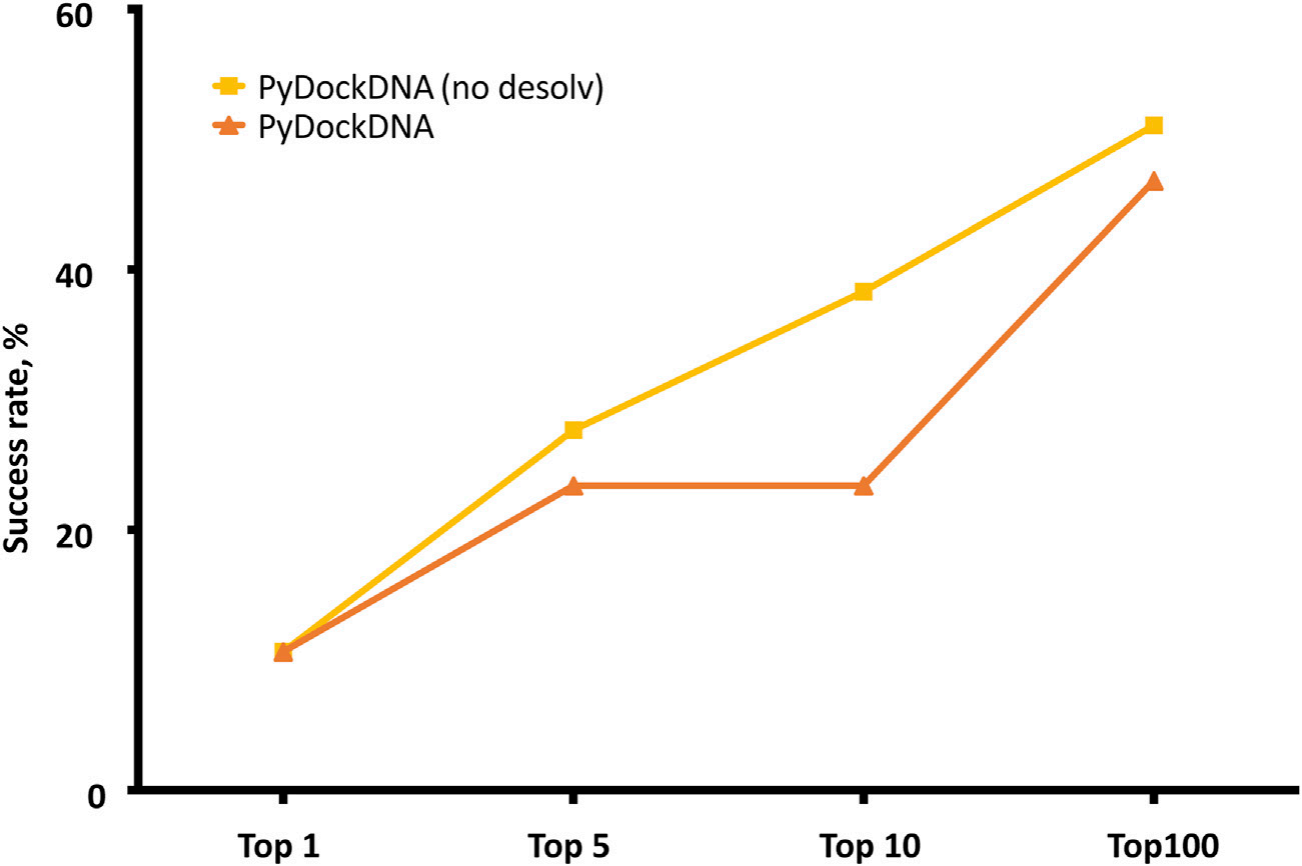
Predictive performance for the top *N = 1, 5, 10, 100* models of pyDockDNA (with and without desolvation) on the protein-DNA docking benchmark.

The pyDockDNA web server has been tested on the 47 cases of a previously reported protein-DNA docking benchmark (see Methods). It is known that using different randomly rotated input structures can slightly affect docking predictions of FFT-based docking protocols as in FTDOCK, because this can modify the mapping of the atom positions on the 3D grids (Garzon et al., 2009; Pallara et al., 2016). To check for convergence, we applied pyDockDNA to 10 different random rotations of the initial input structures for each benchmark case and computed the predictive success rates for the results obtained from each randomly rotated input structures. The results indicate even more differences in the predictive values than previously reported for protein-protein docking (Supplementary Table S1). For instance, the success rates for the top 10 models ranged from 12.8% to 21.3%. Therefore, for a more robust evaluation, we merged the results of all 10 docking executions and clustered the obtained docking models to remove similar orientations (see Methods). Figure 2 shows the predictive success rates of the cluster representatives resulting from merging these 10 docking runs. The predictive success for the default pyDock scoring function (including parameters for nucleotide atoms, see Methods) are better than those obtained for the individual docking runs, which means that increasing sampling variability when using different random initial rotations, followed by redundancy removal with clustering, have improved the docking results.

We further analyzed whether a scoring function previously developed for protein-protein docking was really optimal for protein-DNA docking, since for the latter, electrostatics energy term is expected to have a larger contribution to binding energy due to the higher overall charge of DNA molecules. Moreover, desolvation atomic parameters were previously derived for protein-protein docking in pyDock, but they were not specifically optimized here for nucleotide atoms. To analyze the role of desolvation in protein-DNA scoring, we rescored the generated docking models with the pyDockDNA scoring function but excluding desolvation energy. This greatly improved the success rates, as the curve *pyDockDNA (no desolv)* shows in Figure 2. This indeed indicates that desolvation is not really needed for the scoring of the protein-DNA docking models generated by FFT-based sampling, perhaps because the parameters have not been yet optimized for nucleotide atoms, or because electrostatics is more relevant in protein-DNA interactions than in protein-protein complexes, as above discussed. We tested other solvation parameters for protein-DNA reported in the literature (Kagawa et al., 1989), but the docking results did not improve (further work is needed on the optimization of these parameters in search of a better desolvation for protein-DNA).

In addition, we have also tried other combinations of energy terms, for instance, increasing the factor for van der Waals to 1.0 (we previously found that geometrical complementarity was very important in protein-RNA; (Pérez-Cano et al., 2016), or removing desolvation and van der Waals terms from the scoring function to test the relevance of elecrostatics scoring alone, but none of these new combined scoring functions improved the prediction rates (Supplementary Figure S1).

**FIGURE 3:**
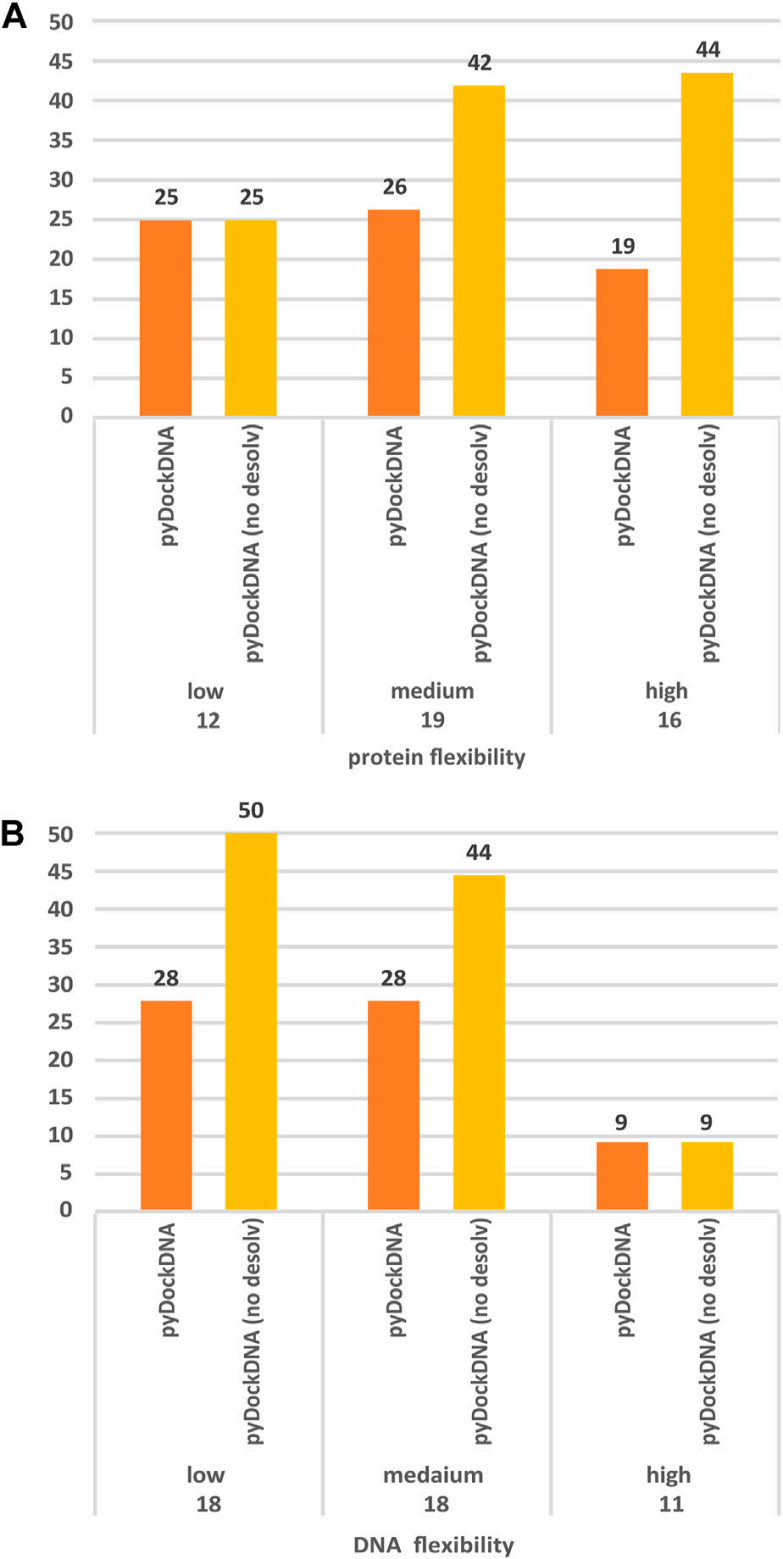
Predictive performance for the top 10 models of pyDockDNA (with and without desolvation) on the protein-DNA docking benchmark when cases are grouped according to **(A)** protein flexibility (low: RMSD <1 Å; medium: 1Å ≤ RMSD <3 Å; high: RMSD ≥3 Å), and **(B)** DNA flexibility (low: RMSD <3 Å; medium: 3 Å ≤ RMSD <5 Å; high: RMSD ≥5 Å). See more details about flexibility definition in main text.

In a rigid-body docking approach as pyDock, it is known that protein flexibility upon binding is perhaps the most determinant factor for docking success. To further analyze whether the docking performance of pyDockDNA is affected by the flexibility of the protein or DNA input molecules during the complex formation, we have grouped the docking results on the protein-DNA docking benchmark according to the flexibility of the protein or the DNA, that is, based on the RMSD between the unbound molecules and the corresponding ones in the complex. Regarding protein flexibility, in order to make groups of similar size, we defined these three categories: low (unbound-bound RMSD <1 Å), medium (1 Å ≤ unbound-bound RMSD <3 Å) and high (unbound-bound RMSD ≥3 Å) flexible cases. As for DNA flexibility, we defined these three categories: low (unbound-bound RMSD <3 Å), medium (3 Å ≤ unbound-bound RMSD <5 Å) and high (unbound-bound RMSD ≥5 Å) flexible cases. The results are shown in Figure 3. We can observe that the docking predictive performance does not worsen when protein flexibility is higher (actually, for pyDockDNA with no desolvation, success rates increase when protein flexibility is medium or high). However, we can see that the docking performance for highly flexible DNA molecules is dramatically low. We should note that in this benchmark, proteins in general show smaller flexibility (average unbound-bound RMSD 2.6 Å) as compared to DNA (average 4.2 Å). In addition, due to the different RMSD cut-off values used to define the flexibility groups for proteins and for DNA, the unbound-bound RMSD values for the group of high flexible proteins (average 4.8 Å) are much smaller than for the group of high flexible DNA (average 7.8 Å), which could explain the much worse predictive rates in the latter.

### Application to external case studies

For further testing, we have applied pyDockDNA to a set of ten additional protein-DNA cases (Table 1) where the structures for the complex and the unbound protein were available at PDB, and the unbound DNA was modelled in canonical B-DNA conformation (see Methods).

For each case study, we performed a single pyDockDNA execution on the randomly rotated unbound protein and DNA structures. This represented a realistic scenario, since the pyDockDNA server only provides results for a docking execution (randomly rotated input structures should be provided to the server in independent executions for a more thorough docking study similar to the benchmark performance analysis above shown). Overall, we obtained predictive success rates of 10% (for the top 10 models) and 30% (for the top 100 models) when using pyDockDNA scoring function, and 10% and 60% (for the top 10 and 100 models, respectively), when using pyDockDNA without desolvation. Given the small number of cases of these additional set, these values are within the expected range according to the larger docking benchmark set.

**FIGURE 4:**
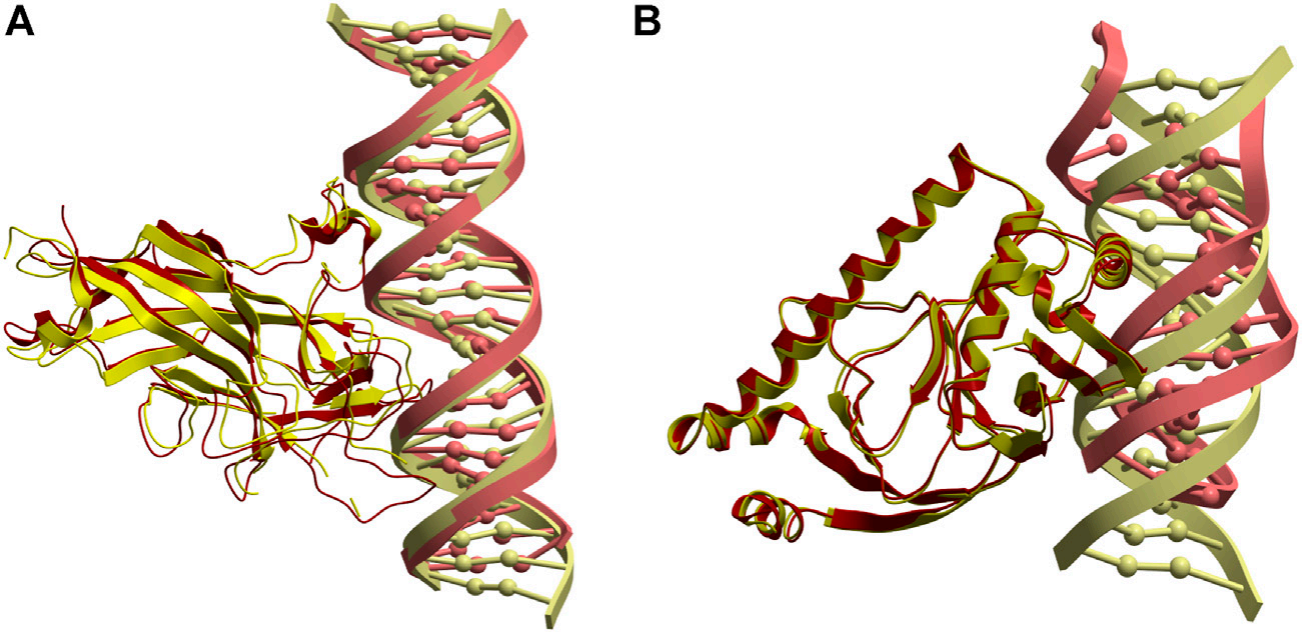
Application of pyDockDNA to case studies. **(A)** Near-native model (in yellow) obtained by pyDockDNA docking between a modelled 22bp DNA (receptor) and Ebf1 (ligand). This model was ranked 5 with pyDockDNA (no desolvation) scoring function and has L-RMSD 3.33 Å with respect to the reference (PDB 3MLO; in red). **(B)** Reasonable model (in yellow) obtained by pyDockDNA docking between the catabolite gene activator protein (receptor) and a modelled 11bp DNA (ligand). This model was ranked 5 with pyDockDNA (either with desolvation or with no desolvation) scoring function and has L-RMSD 10.76 Å with respect to the reference (PDB 1O3R; in red).

The most successful case is the complex between the DNA binding domain of Early B-cell Factor 1 (Ebf1) bound to a 22bp DNA (PDB 3MLO), where a near-native docking model (L-RMSD 3.33 Å with respect to the reference) is found with rank five when using pyDockDNA (no desolvation) scoring function (Figure 4A). When using pyDockDNA (including desolvation) scoring function, this docking model is ranked 6, so it is still within top 10 models. This case has low-flexible protein but high-flexible DNA.

Another case is the complex between the catabolite gene activator protein and a 11bp DNA (PDB 1O3R), where we found an almost acceptable docking model (L-RMSD 10.76 Å with respect to the reference) with rank 5, when using pyDockDNA either including solvation or not (Figure 4B). This case has also low-flexible protein but medium-flexible DNA. Incidentally, if this case were considered acceptable, the success rate for the top 10 would be 20%. However, these percentage values are perhaps not very meaningful considering the low number of cases in this external test set. Interestingly, when using van der Waals term with weighing factor 1.0 (instead of the default factor in pyDock and pyDockDNA, that is 0.1), we find near-native solutions in three more cases, in addition to 3MLO: 1) 5JLT (L-RMSD 7.08 Å) with rank one when using desolvation; 2) 2NTC (L-RMSD 7.25 Å) with rank three without using desolvation, and 3) 2PI0 (L-RMSD 6.63 Å) with rank 3 and 2, with or without desolvation, respectively. Therefore, for half of these external case studies, we found near-native docking models within the top 10 models with pyDockDNA, using different variants of the scoring function.

In summary, we present here the pyDockDNA web server to model protein-DNA complexes, which implements a docking method based on pyDock, with new scoring parameters for DNA. We have evaluated the performance on unbound proteins and modelled DNA molecules in canonical B-DNA conformation, using a known protein-DNA docking benchmark. The results show near 40% success rate for the top 10 models when using the pyDockDNA (no desolvation) scoring function, after merging the results from 10 docking executions using different randomly rotated initial structures, and clustering the models to remove redundant ones. The method has been applied to external case studies, with similar predictive performance.

## Data Availability

The original contributions presented in the study are included in the article/Supplementary Material, further inquiries can be directed to the corresponding author.
