# Supplementary material for: pyDockDNA: A new web server for energy-based protein-DNA docking and scoring
Source: Front Mol Biosci. 2022 Oct 6;9:988996. doi: 10.3389/fmolb.2022.988996 (PMC9582769; doi:10.3389/fmolb.2022.988996)
Supplement: Supplementary file 1 [file DataSheet2.PDF]

Table S1: pyDockDNA docking performance for protein-DNA docking benchmark using 10 random rotations of input structures for each case.

	% success rates			
**rotation**	**top 1**	**top 5**	**top 10**	**top 100**
#1	8.5	12.8	17.0	44.7
#2	4.3	14.9	21.3	44.7
#3	8.5	12.8	17.0	44.7
#4	4.3	8.5	12.8	36.2
#5	6.4	14.9	19.1	53.2
#6	6.4	12.8	17.0	42.6
#7	8.5	17.0	19.1	53.2
#8	4.3	8.5	12.8	42.6
#9	4.3	12.8	14.9	46.8
#10	4.3	8.5	12.8	40.4
average	*6.0*	*12.4*	*16.4*	*44.9*
