# Supplementary material for: pyDockDNA: A new web server for energy-based protein-DNA docking and scoring
Source: Front Mol Biosci. 2022 Oct 6;9:988996. doi: 10.3389/fmolb.2022.988996 (PMC9582769; doi:10.3389/fmolb.2022.988996)

Figure S1. Predictive performance for the top *N=1, 5, 10, 100* models of pyDockDNA and different combinations of scoring terms on the protein-DNA docking benchmark.

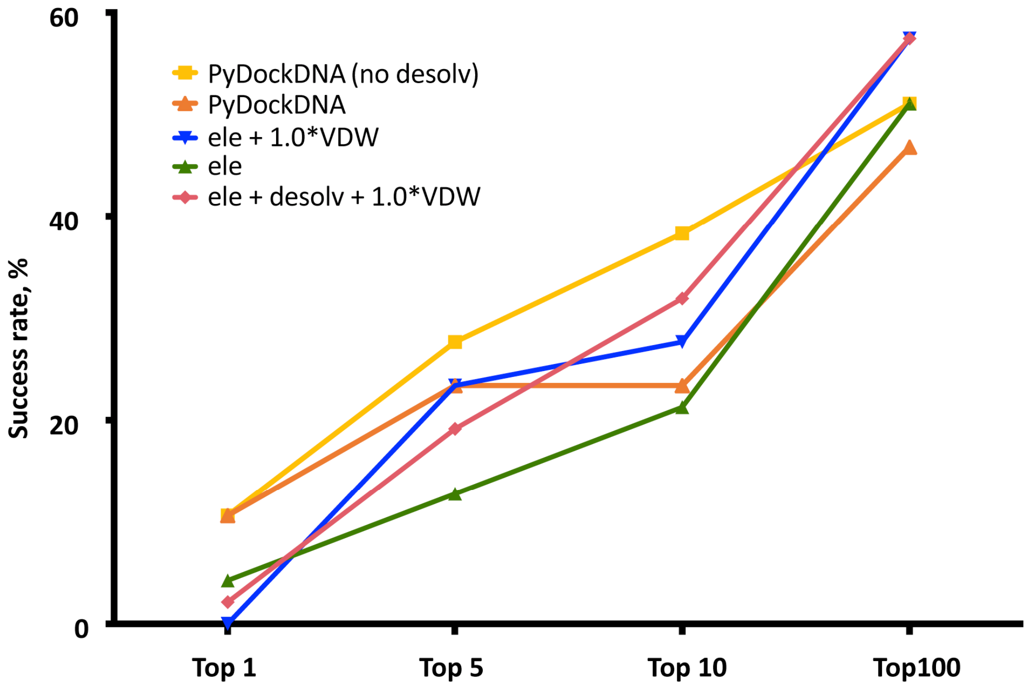

Supplement: Supplementary file 2 [file DataSheet1.PDF]
